# Applications of Gene Editing in Chickens: A New Era Is on the Horizon

**DOI:** 10.3389/fgene.2018.00456

**Published:** 2018-10-09

**Authors:** Hicham Sid, Benjamin Schusser

**Affiliations:** Department of Animal Sciences, Reproductive Biotechnology, School of Life Sciences Weihenstephan, Technical University Munich, Freising, Germany

**Keywords:** chicken, CRISPR/Cas9, transgenic, knockout, Diseases, Immunoglobulins

## Abstract

The chicken represents a valuable model for research in the area of immunology, infectious diseases as well as developmental biology. Although it was the first livestock species to have its genome sequenced, there was no reverse genetic technology available to help understanding specific gene functions. Recently, homologous recombination was used to knockout the chicken immunoglobulin genes. Subsequent studies using immunoglobulin knockout birds helped to understand different aspects related to B cell development and antibody production. Furthermore, the latest advances in the field of genome editing including the CRISPR/Cas9 system allowed the introduction of site specific gene modifications in various animal species. Thus, it may provide a powerful tool for the generation of genetically modified chickens carrying resistance for certain pathogens. This was previously demonstrated by targeting the Trp38 region which was shown to be effective in the control of avian leukosis virus in chicken DF-1 cells. Herein we review the current and future prospects of gene editing and how it possibly contributes to the development of resistant chickens against infectious diseases.

## Introduction

The chicken represents an important source of protein worldwide and a valuable model for the study of developmental biology in vertebrates (Yasugi and Nakamura, 2000; Speedy, 2003). Chickens are constantly exposed to a plethora of pathogens threatening animal welfare as well as human health (Perdue and Swayne, 2005; Humphrey, 2006). Viral pathogens such as influenza A viruses can be transmitted to humans leading to death (Gao et al., 2013). Furthermore, bacterial agents such as *Campylobacter jejuni* and *Salmonella enteritidis* cause food borne illnesses in humans associated with digestive symptoms (Bryan and Doyle, 1995). More recently, using genetically modified chickens as a model for various research areas like developmental biology, immunology, physiology and neurology is gaining importance in the avian research community (Mozdziak and Petitte, 2004; Stern, 2004, 2005). In addition, there is an increasing interest to generate genetically modified chickens resistant to specific pathogens, benefiting from the availability of gene manipulation techniques. This review focuses on the advances made in gene editing in chickens and the future perspectives including the generation of specific-pathogen-resistant birds.

## State of the art

Genetically modified animals have significantly contributed to our understanding of different aspects related to immunity, infectious diseases, neurology, behavior, and developmental biology (Yeh et al., 2002; Lyall et al., 2011; Lalonde et al., 2012; Pinkert, 2014; Park et al., 2017b). While mice were the first animals to be genetically modified (Costantini and Lacy, 1981; Gordon and Ruddle, 1981), pronuclear DNA microinjections allowed the introduction of foreign DNA leading to genetic modifications in livestock including rabbits, sheep and pigs (Hammer et al., 1985). Although this method was used for a long time, it did not allow the induction of targeted gene modifications and had the disadvantage of generating random integrations (Perleberg et al., 2018). The generation of knockout (KO) animals was achieved for the first time by gene targeting in embryonic stem cells (ES) (Evans and Kaufman, 1981; Thomas and Capecchi, 1987). Though the induction of the KO was successful, it had the disadvantage of low efficiency (Thomas and Capecchi, 1987). Due to the absence of true ES lines from farm animals and no solid evidence of germline transmission (Talbot and Blomberg, 2008; Soto and Ross, 2016), stable transfection of sheep somatic cells with human factor IX and neomycin resistance followed by nuclear transfer was the alternative to express foreign DNA in livestock (Schnieke et al., 1997) and afterwards for gene targeting (McCreath et al., 2000). At this time, the generation of KO livestock animals was possible by combining somatic cell nuclear transfer (SNTC) and homologous recombination (Lai et al., 2002; Nottle et al., 2007). The laborious procedure of these methods and the low efficiency for generating targeted KO was improved by homologous recombination (Houdebine, 2002) along with different nucleases (Carlson et al., 2012). The transcription activator-like effector nucleases (TALENs) are composed of series of repeats fused to non-specific FokI-cleavage domains that induce double- stranded DNA breaks upon dimerization (Gaj et al., 2013). More recently the Clustered Regularly Interspaced Short Palindromic Repeats (CRISPR)/Cas9 system made the process of specific DNA-targeting easier by using single guide RNAs (sgRNAs) (Jinek et al., 2012; Ran et al., 2013; Hsu et al., 2014). CRISPR/Cas9 is an adaptive immune system found in bacteria and archaeal species and uses small-non coding RNAs to guide the Cas9 nuclease to target sites resulting in DNA double-break (Jinek et al., 2012).

In comparison to mammals, difficulties were always associated with the generation of genetically modified chickens due to the complex structure of the chicken zygote (Mozdziak and Petitte, 2004) and the different organization of the chick embryo compared to mammals (Stern, 1990). Over the past 30 years, different research groups paved the way for the generation of genetically modified chickens. Efforts were focused on the stable genomic integration of transgenes and obtaining the highest efficiency of germline transmission. While Pettite and colleagues described the transfer of stage X embryo cells that led to germline transmission, it was not possible to genetically modify these cells and to re-introduce them as germline competent cells into the chicken embryo (Petitte et al., 1990). Although ES were shown to provide a valuable tool for the generation of transgenic mice (Kanatsu-Shinohara et al., 2003), no evidence of germline transmission using chicken ES was reported. Transferred chicken ES cells only contributed to somatic tissue but not to the germline.

The first genetically modified chicken was generated by the insertion of retroviral foreign DNA delivered by avian leukosis virus that was successfully integrated to the germline (Salter et al., 1987). The retroviral vector was injected into the yolk sac near to the developing blastoderm. Since then, various viral vectors have been used to generate genetically modified chickens (Hughes et al., 1986; Bosselman et al., 1989; Salter and Crittenden, 1989; Harvey and Ivarie, 2003; Mozdziak et al., 2003). Drawbacks of viral vectors, such as the replication of deficient viral particles and risks of recombination with wild type viruses, were avoided by plasmid-DNA microinjection into the chicken zygote (Love et al., 1994). The microinjection was done in the germinal disk and led to the generation of transgenic chickens expressing neomycin resistance and a reporter gene *lacZ* (Love et al., 1994). A total of 5.5% of the generated chicks survived to sexual maturity and later on, one rooster gave 3.4% transmission to his offspring (Love et al., 1994). The germline transmission of integrated transgenes was improved with lentiviral vectors (McGrew et al., 2004). McGrew and colleagues showed the possibility of transduction with lentiviral vectors in G0 birds. Founder cockerels were injected with different plasmids carrying different reporter genes including *LacZ* and eGFP (McGrew et al., 2004). Lentiviral vectors were injected into the subgerminal cavity of newly laid eggs. Ten of the founder males transmitted 4-45% of the foreign DNA to their offspring (McGrew et al., 2004). Lentiviral vectors offered for the first time the possibility to generate genetically modified chickens with a decent germline transmission efficiency. Nevertheless, the size of the transgene was still limited and precise edits were not possible.

Furthermore, the *in ovo* injection of the avian retroviral vector RCAS (replication-competent avian sarcoma-leukosis virus with a splice acceptor) carrying enhanced fluorescent protein (eGFP) into unincubated (stage X) blastoderms resulted in stable and widespread expression of eGFP in the embroys. Even though the gonads showed eGFP expression PGCs were eGFP negative indicating viral silencing (Smith et al., 2009).

Like in mammals, chicken primordial germ cells (PGCs) are precursors of gametes and a key element for sperm and oocystes development. At the early hours of embryonic development, PGCs are found in the germinal crescent and migrate afterwards (50–55 h) to the gonads (Kim et al., 2010; Kang et al., 2015) in order to produce sperm and oocystes upon sexual maturity (Fujimoto et al., 1976). The migration of PGCs was found to be greatly influenced by the chemokine stromal cell-derived factor-1 (SDF-1/CXCL12) and its receptor C-X-C chemokine receptor type 4 (CXCR4) (Stebler et al., 2004; Lee et al., 2017c).

The ability to culture PGCs was a milestone in the process of generating transgenic chickens. Genetic modification of PGCs and their subsequent reintroduction into the embryonic vasculature resolved many issues and problems observed with previously established methods. Van de Lavoir and colleagues used BRL or STO feeder cells to cultivate PGCs for up to 217 days. PGCs were shown to retain the germline characteristics by analyzing various germline markers including the chicken vasa homolog (CVH) and were cryoconserved using conventional techniques (Van De Lavoir et al., 2006). PGC-culture was optimized afterwards by Whyte and colleagues that developed feeder and serum free culture conditions that took into consideration the signaling pathways necessary for avian germ cell self-renewal (Whyte et al., 2015). The work of van de Lavoir and colleagues revealed that foreign DNA can be inserted in the genome of PGCs and cells were still restricted to the germline (Van De Lavoir et al., 2006; Leighton et al., 2008). Male PGCs were cultured for a duration between 35 and 110 days during which they were transfected with a construct coding for eGFP and subsequently injected into the vasculature of White Leghorn embryos [stage 13–15 Hamburger and Hamilton (H&H)](Van De Lavoir et al., 2006). Interestingly, the long term culture of PGCs did not influence their ability to colonize the gonads after insertion of foreign DNA, which allowed afterwards the generation of several transgenic chicken lines (Van De Lavoir et al., 2006, 2012; Macdonald et al., 2012). Leighton and colleagues gave new insights about increasing the efficiency of foreign DNA insertion in PGCs mediated by phiC31 integrase that catalyzes site-specific recombination between attB and pseudo attP sites in the chicken genome and increases transgene integration (Leighton et al., 2008).

Lu and colleagues indicated that the *piggyBac* transposon can be efficiently integrated into the genome of chicken embryo during development via electroporation (Lu et al., 2009). The transfection of PGCs with *piggyBac* transposon greatly enhanced the integration frequency of foreign DNA into the chicken genome and resulted in the generation of genetically modified chickens (Park and Han, 2012; Glover et al., 2013). In contrast, the injection of *piggyBac* transposon into the subgerminal cavity of a newly laid egg and subsequent electroporation, resulted in chickens expressing the transgene but no germline transmission was detectable (Liu et al., 2013). At the same time, Tyack and colleagues successfully developed a method for the direct transfection of circulating PGCs using Lipofectamine 2000 in combination with Tol2 transposon and transposase plasmids (Tyack et al., 2013). The plasmid contained the pCAGGS promoter driving the expression of eGFP. Tyack and colleagues found that 5/11 roosters expressed the miniTol DNA in their semen and two of them gave about 1.5% germline transmission (Tyack et al., 2013). This method substantially reduced the time needed for the *in vitro* isolation and gene manipulation of PGCs; however, it did not increase the germline transmission in G0 (Tyack et al., 2013). In addition, it does not allow clonal selection of PGCs and may result in birds with random integrations of the same transgene. Nevertheless, it is an effective method to produce genetically modified chickens as shown by various publications (Tyack et al., 2013; Lambeth et al., 2016a,b).

The possibility to culture and genetically modify chicken PGCs without losing germline competence made it possible to perform precise gene deletions and integrations in the chicken genome. Specific gene locus KO chickens were generated by Schusser and colleagues via gene targeting by homologous recombination in chicken PGCs (Schusser et al., 2013a, 2016). In the case of targeted immunoglobulin heavy chain J segment, a total of 7 from 27 PGC clones (28%) had a correctly targeted event which reflected a high efficiency comparable to mouse ES cells (Schusser et al., 2013a). Similar efficiency was obtained after targeting the immunoglobulin light chain locus in chicken PGCs. After successful targeting of the immunoglobulin heavy or light chain in chicken PGCs, resulting clones were injected into H&H stage 13–15 embryos in order to generate germline chimeras. Germline transmission rates varied between 0.1 and 48% depending on the used PGC clone (Schusser et al., 2013a, 2016). Resulting homozygous immunoglobulin heavy chain J segment knockout birds showed a depletion of peripheral B cells and antibodies and were the first non-mammalian vertebrates harboring a knockout produced by homologs recombination. In order to perform gene knockouts by homologs recombination in PGCs, isogenic DNA is needed since mismatches in the homology regions are not tolerated (Schusser et al., 2016).

Since PGCs are precursors of sperm, researchers suggested that roosters could be used as recipient for exogenous transfer of genetically-modified PGCs which may improve the germline transmission rate (Trefil et al., 2017). Chicken embryos and adult roosters were chemically or physically sterilized to create a surrogate for external PGC donors (Trefil et al., 2006; Nakamura et al., 2008, 2010; Ghadimi et al., 2017). Nakamura and colleagues partially sterilized chicken embryos by injecting Busulfan into the yolk of fertile eggs before incubation; this led to a significant reduction of endogenous PGCs. Authors demonstrated that the sterilized embryos can be used for exogenous transfer of PGCs resulting in high efficiencies of germline transmission (Nakamura et al., 2008, 2010). Early experiments performed by Trefil and colleagues provided an alternative for chemical sterilization and concluded that repeated gamma irradiation leads to sterilization of roosters (Trefil et al., 2006). Performing injection of donor spermatogonial cells led to reestablishment of male function in 50% of the roosters only 5 weeks after injection (Trefil et al., 2006). Spermatogenesis was restored 4 weeks later in the case of PGC-transplantation compared to spermatogonial cells; however, PGCs exhibited higher efficiency in repopulating the seminiferous epithelium (Trefil et al., 2006, 2017). This was very beneficial in the case of transplantation of genetically modified PGCs into mature roosters after complete irradiation (Trefil et al., 2017). Male fertility was reestablished after the transplantation of GFP- or mCherry-expressing PGCs and resulted in almost 100% germline transmission (Trefil et al., 2017). The prominent advantage of this method is the certainty of the germline transmission and the low number of animals used in the experiment; hence reducing time and costs for testing high number of chimeric roosters. Although using gamma irradiation to sterilize roosters was as efficient as in mice, recent findings in pigs suggested that the knockout of *NANOS2*, like in mice, results in specific germline ablation with preserved testicular development (Park et al., 2017a); therefore it was suggested that *NANOS2* KO pigs may serve as a surrogate for transplantation of donor spermatogonial cells (Park et al., 2017a). Even though the importance of *NANOS2* in the transformation of ES into germ cells is well determined, little is known about its function in chickens. The most important steps made in the process of generating genetically modified chickens are summarized in Table 1.

**Table 1:** Important steps for the generation of genetically modified chickens.

**Year of publication**	**1987**	**1994**	**2004**	**2006**	**2013**	**2013**	**2015**	**2016**	**2017**	**2017**
Contribution	Generation of the first genetically modified chicken using retroviral vectors	Generation of the first genetically modified chicken by DNA microinjection	Transgenic chickens using lentivirus carrying a transgene	Isolation and long term culture of PGCs followed by transfection and generation of eGFP transgenic chickens	Targeted gene editing in PGCs and generation of the first gene knockout chickens	*In vivo* transfection of PGCs using Lipofectamine 2000 complexed with Tol2 transposon and transposase plasmids	Development of feeder and serum-free PGC cultures	CRISPR-mediated homology directed repair targeting the immunoglobulin heavy chain locus in PGCs.	TALEN-mediated gene targeting of PGCs	Restoration of male fertility by transplantation of genetically modified PGCs
References	Salter et al., 1987	Love et al., 1994	McGrew et al., 2004	Van De Lavoir et al., 2006	Schusser et al., 2013a	Tyack et al., 2013	Whyte et al., 2015	Dimitrov et al., 2016	Taylor et al., 2017	Trefil et al., 2017

## Gene editing in avian cell lines

The unavailability of fully transgenic chickens for a long time encouraged the development of alternative methods based on *in vitro* cell culture systems. *In vitro* studies helped to provide valuable data regarding host susceptibility to specific pathogens and the role of specific genes during host-pathogen interactions. DT-40 cells, an avian leukosis virus induced bursal B- cell lymphoma line, was extensively used to investigate B cell immunology, cell cycle regulation, gene conversion and apoptosis (Uckun et al., 1996; Arakawa et al., 2001; Harris et al., 2002; Arakawa and Buerstedde, 2004). A large number of DT-40 mutants were generated to understand B cell biology and were reviewed elsewhere (Arakawa and Buerstedde, 2004). For instance, studies based on DT-40 cells proved that the activation-induced cytidine deaminase (AID) triggers immunoglobulin gene diversification by gene conversion (Buerstedde et al., 1990; Kim et al., 1990). Furthermore, Szüts and colleagues used mutant DT-40 cells to demonstrate the role of RAD18 in DNA repair and the completion of gene conversion (Szüts et al., 2006). Interestingly, Schusser and colleagues replaced the immunoglobulin light and heavy chain loci in DT-40 cells with human immunoglobulin light and heavy chain loci; this led to the expression of chimeric IgM with human variable regions and chicken constant regions (Schusser et al., 2013b). The later cell line provides a model to study the diversification of the human variable region by gene conversion and somatic hypermutations in chickens. Antigen receptor analysis were performed by deep sequencing confirming that the host machinery in DT-40 cells diversified the integrated human V genes (Leighton et al., 2015).

A different established model for examining gene function in chickens is the Douglas Foster (DF-1) cells, an immortalized chicken fibroblast cell line (Foster, 1998). Recent studies used DF-1 cells to investigate host-pathogen interactions of several avian pathogens with the avian host; this included influenza A viruses, Newcastle disease virus, infectious bursal disease virus and retroviruses (Huang et al., 2003; Lee et al., 2008; Cheng et al., 2015; Hui and Leung, 2015). The overexpression of different avian genes in DF-1 cells helped to examine their role in the innate immunity against viral pathogens (Shao et al., 2014; Cheng et al., 2015; Xu et al., 2015). A well-known tool for the overexpression of various genes is the retroviral vectors derived from the SR-A strain of Rous sarcoma virus (RCAS). The RCAS system is known for its stable transduction in developing chicken embryo and cell culture (Fekete and Cepko, 1993; Bell and Brickell, 1997). Reuter and colleagues used DF-1 cells for the overexpression of the chicken IFN-α and IFN-λ (Reuter et al., 2014). The overexpression of IFN-λ in DF-1 cells did not cause substantial viral resistance against influenza A viruses H1N1, H7N1, and vesicular stomatitis virus (VSV) (Reuter et al., 2014) which suggested that DF-1 cells have weak antiviral activity of IFN-λ (Karpala et al., 2008). This was not the case for IFN-α where the overexpression led to protection against previously mentioned viruses (Reuter et al., 2014). In addition, DF-1 cells were useful to study the function of foreign genes in chicken including intracellular pattern recognition receptor such as the retinoic inducible resistant gene (RIG-I). RIG-I from duck and goose was overexpressed in DF-1 cells and its protective effect against influenza A viruses and infectious bursitis virus (IBDV) was investigated (Barber et al., 2010; Sun et al., 2013; Shao et al., 2014). The overexpression of duck RIG-I in DF-1 cells reduced viral replication and upregulated virus-induced apoptosis following IBVD- and H9N2 influenza virus infections (Shao et al., 2014). Interestingly, the knockdown of the chicken ANP32A, a nuclear protein implicated in mRNA transport and cell death (Reilly et al., 2014), reduced the activity of different avian influenza polymerases in DF-1 cells. This indicated that avian influenza virus polymerases are more adapted to avian *ANP32A* and proposed this gene as target for antiviral drugs (Long et al., 2016). Furthermore, the overexpression of the chicken GADD45β, a protein associated with cell growth control, apoptotic cell death, and the cellular response to DNA damage (Zazzeroni et al., 2003), helped to limit viral infection which could be used in the future as potential treatment for avian leukosis virus (ALV)-J infections (Zhang et al., 2016).

ALV is one of the most commonly occurring retroviruses in chickens. It induces a variety of neoplastic lesions causing losses in the productivity of affected chicken flocks (Fadly, 2000). Maas and colleagues confirmed that DF-1 cells are much more suitable than primary chicken fibroblasts (CEFs) to study host-pathogen interactions of leukosis viruses with avian cells (Maas et al., 2006). ALV was detected earlier in DF-1 cells and the infection was associated with apparent cytopathogenic effect (CPE) compared to infected-CEFs that had no apparent CPE (Maas et al., 2006). Mutations responsible for the inhibition of ALV subgroup A cell-entry were identified (Klucking et al., 2002) and consisted of four base pairs insertion and one base pair substitution in tumor virus locus A (*tva*) (Klucking et al., 2002). On the other side, only one base pair substitution in the cysteine-rich domain (CRD) of *tvb* receptor led to reduced susceptibility of DF-1 cells to infection with ALV subgroup B (Klucking et al., 2002; Reinisová et al., 2008). Interestingly, subgroup J ALV (ALV-J) uses the multimembrane-spanning cell surface protein, the chicken Na+/H+ exchanger type 1 (NHE1), as a receptor. The attachment of the virus to the receptor is crucial to initiate the infection (Barnard et al., 2006). Kučerová and colleagues used mutagenesis to introduce changes in the subgenic fragment of NHE1 (Kučerová et al., 2013); authors described the functional importance of tryptophan reside at position 38 (Trp38) for virus entry (Kučerová et al., 2013).

The rapid development of gene editing tools such as CRISPR/Cas9 rendered cell culture systems much more useful by easily targeting different genes. Precise gene editing of the chicken *NHE1* gene using CRISPR/Cas9 system led to resistance of DF-1 cells against ALV-J infection (Lee et al., 2017a). The precise genome editing of NHE1 was performed via homologs directed repair (HDR) that combined CRISPR/Cas9 vectors with single-stranded oligodeoxynucleotide (ssODNs). Authors confirmed previous observations mentioning that mutation in the Trp38 are detrimental for ALV-J infection (Kučerová et al., 2013). On the other side, non-homologous end joining repair (NHEJ) was also established in DF-1 cells. Targeting the tumor virus locus B gene, which serves as entry receptor for ALV subgroup B, resulted in frameshift mutations leading to a KO of the *tvb*-receptor in DF-1 cells, which conferred resistance against ALV-B (Lee et al., 2017b). Abu-Bonsrah and colleagues targeted a wide range of genes in DF-1 cells such as *DROSHA, DICER, MBD3, KIAA1279, CDKN1B, EZH2, HIRA, TYRP1, STMN2, RET*, and *DGCR*, that play a role in embryonic development and pathogenesis of embryonic diseases (Abu-Bonsrah et al., 2016). Efficiency of inducing mutations was analyzed by T7E1 assay. The efficiency ranged between 20 and 65% in DF-1 cells (Abu-Bonsrah et al., 2016). Similar results were obtained after knocking out *KIAA1279*- and *CDKN1B*-genes in DT-40 cell-line via electroporation (Abu-Bonsrah et al., 2016). Likewise, Bai and colleagues gave more insights about the efficiency of CRISPR/Cas9 in DF-1 cells by studying gene editing in the presence and the absence of puromycin antibiotic selection (Bai et al., 2016). Three genes including peroxisome proliferator-activated receptor-γ *(PPAR-*γ), ATP synthase epsilon subunit (*ATP5E*), and ovalbumin (*OVA*) were targeted with CRISRP/Cas9 vectors. T7E assay indicated that puromycin selection increased mutation rate in the previously mentioned genes from 0.75, 0.5, and 3.0%, to 60.7, 61.3, and 47.3%, respectively (Bai et al., 2016).

## Gene editing in the chicken embryo

The chicken embryo is a well-established model to study developmental processes, gene functions and host-pathogen interactions (Darnell and Schoenwolf, 2000; Chesnutt and Niswander, 2004; Schecterson et al., 2012). Over the last decades, different methods were established to genetically manipulate chicken embryos including electroporation of foreign DNA constructs, transduction with retroviruses and recently the combination of previous known methods with CRISPR/Cas9 system (Gandhi et al., 2017).

For example, Luo and colleagues established a protocol based on *ex ovo* electroporation of 3.5 days old chicken embryos for the overexpression of Cad7 and eGFP (Luo et al., 2012). This method provided accessibility of different embryonic parts for the electroporation, which are not easily reachable when the embryo is still inside the egg (Luo et al., 2012). Similarly, *in ovo* electroporation of the embryonic auditory brainstem was previously established (Lu et al., 2017). Plasmids of interest were successfully integrated into the nucleus magnocellularis and nucleus laminaris. Authors indicated the possibility of drug inducible gene expression which was confirmed in the presence of doxycycline (Lu et al., 2017).

A well-established tool for foreign DNA integration is the RCAS-system. Using RCAS in the chicken embryo model indicated that vector proteins and inserted transgenes were mainly detectable in the skin, blood vessels and heart (Sato et al., 2002; Kothlow et al., 2010). Several studies deduced the efficacy of RCAS-system in the case of foreign DNA integration and gene overexpression in chicken embryos (Bell and Brickell, 1997; Sato et al., 2002; Kothlow et al., 2010; Schusser et al., 2011; Reuter et al., 2014). This system is very useful to study the specific function of relevant genes for the innate immunity, particularly during the interaction with influenza A viruses. RCAS vectors expressing various *Mx* gene isoforms were used for transduction of CEFs. Four days post-transfection, CEFs expressing the retrovirally transduced Mx proteins were injected in the yolk sac of 3 days-old fertilized eggs (Schusser et al., 2011). The overexpression of different Mx isoforms in embryonated eggs did not protect against influenza A virus infection, which was in agreement with the results obtained from chicken fibroblasts (Schusser et al., 2011). In addition, the role of IFN-λ was previously investigated by the generation of mosaic chicken embryos overexpressing chicken IFN-λ (Reuter et al., 2014). Generated embryos exhibited lower viral titers upon challenge with influenza A viruses, NDV Herts-33, or IBV M-41 via the allantoic cavity by at least four log_10_ units compared to inoculated eggs with empty RCAS vector (Reuter et al., 2014). This clearly demonstrated the protective effect of chicken IFN-λ against different viruses (Reuter et al., 2014). Although the IFN-λ overexpression had detrimental effects at early hours post hatch (Reuter et al., 2014), RCAS system was shown to be successful for maintaining transgene expression after hatch (Kothlow et al., 2010).

A similar system based on gene transfer mediated by lentiviral vectors was described in embryonated eggs (Hen et al., 2012). Usefulness of lentiviral vectors in developmental biology was previously reviewed elsewhere (Stern, 2004). Lentiviral vectors of feline immunodeficiency virus origin were injected into chorioallantoic membrane (CAM) of 11 days old chicken embryos. The injected lentiviral vectors carried yellow fluorescent protein (YFP) or recombinant alpha-melanocyte-stimulating hormone (α-MSH) genes and they were expressed under the cytomegalovirus (CMV) promoter (Hen et al., 2012). High efficiency of transduction was observed in the liver, which implied that this model could be useful for the study of hormones and enzymes.

The application of gene editing technologies via *in ovo* electroporation of chicken embryos seems to be efficient (Wilson and Stoeckli, 2012). Wilson and Stoeckli used miRNA-based plasmids for knocking down gene expression in the chicken neural tube (Wilson and Stoeckli, 2012). Additionally, Ghandi and colleagues used *ex ovo* electroporation to knockout Pax7 and Sox10, a key transcription factors in the neural crest, leading to loss of their proteins and transcripts (Gandhi et al., 2017). Overall, collected data indicated that *in ovo* gene manipulation of the chicken embryo could be used as a model for the study of different embryonic developmental stages (Gandhi et al., 2017; Lu et al., 2017). High targeting efficiency and the simplicity of CRISPR/Cas9 make it now possible to knockout genes in specific tissues/organs of the developing chicken embryo. This allows the study the gene function during development without generating fully gene edited chicken lines.

## Generation of genetically modified chickens

The generation of genetically modified chickens has wide applications in agricultural and biomedical research (Sang, 1994; Ivarie, 2003; Mozdziak and Petitte, 2004). Benefiting from gene editing technologies and germline transmission of PGCs, new knowledge was brought to light about specific gene functions (Schusser et al., 2013a, 2016), resistant for infectious diseases (Lyall et al., 2011) and the possible preservation of endangered species including the Houbara Bastard (Kang et al., 2008; Wernery et al., 2010; Van De Lavoir et al., 2012). Different methods used for gene editing in chickens and the generated chicken lines were stated earlier in this review. In addition, the worldwide availability of genetically modified chicken lines is summarized in Table 2.

**Table 2:** Worldwide availability of genetically modified chickens.

**Transgenic chicken**	**Affiliation of the research group**	**Country**	**References**
Transgenic chickens carrying a benign defective subgroup A leukosis virus	Avian Disease and Oncology Laboratory, USDA Agriculture Research Service	USA	Salter and Crittenden, 1989; Cao et al., 2015
Transgenic chickens expressing active β-lactamase in the egg white	AviGenics, Inc., Georgia BioBusiness Center, Athens	USA	Harvey et al., 2002
eGFP expressing chickens	The Roslin Institute and Royal Dick School of Veterinary Studies, University of Edinburgh	UK	McGrew et al., 2004
	Department of Physiology, Catholic University of Daegu School of Medicine	South Korea	Kwon et al., 2004
	University of Utah School of Medicine, Department of Neurobiology, and Anatomy	USA	Chapman et al., 2005
	Crystal Bioscience/ Ligand Pharmaceuticals Inc	USA	Van De Lavoir et al., 2006
	Technical University Munich, School of Life Sciences Weihenstephan; Department of Animal Sciences, Reproductive Biotechnology	Germany	Trefil et al., 2017
Hens specifically expressing therapeutic proteins in the oviduct	The Roslin Institute and Royal Dick School of Veterinary Studies, University of Edinburgh	UK	Lillico et al., 2007
Production of transgenic chickens expressing a tetracycline-inducible eGFP gene	Department of Physiology, Catholic University of Daegu School of Medicine, Daegu	South Korea	Kwon et al., 2011
Short-hairpin RNA against Influenza expressing chickens	Department of Veterinary Medicine, University of Cambridge, Madingley Road, Cambridge	UK	Lyall et al., 2011
Transgenic chickens expressing human extracellular superoxide dismutase	Laboratory of Dermatology-immunology, The Catholic University of Korea	South Korea	Byun et al., 2013
Immunoglobulin heavy chain (JH) KO chickens	Crystal Bioscience/ Ligand Pharmaceuticals Inc	USA	Schusser et al., 2013a
Transgenic chickens expressing the human urokinase type-plasminogen activator	Department of Animal Biotechnology, Bio-Organ Research Center, Konkuk University	South Korea	Lee et al., 2013
CSF1R-receptor reporter chickens	The Roslin Institute and Royal Dick School of Veterinary Studies, University of Edinburgh	UK	Balic et al., 2014
Immunoglobulin light chain (IgL) KO chickens	Crystal Bioscience/Ligand Pharmaceuticals Inc	USA	Schusser et al., 2016
Cre-recombinase expressing chickens	Crystal Bioscience/Ligand Pharmaceuticals Inc	USA	Leighton et al., 2016
Ovalbumin and Ovomucoid KO chickens	Biomedical Research Institute, National Institute of Advanced Industrial Science and Technology and Animal Breeding and Reproduction Research Division	Japan	Oishi et al., 2016
Aromatase overexpressing chickens	Department of Anatomy and Developmental Biology, Monash University, Clayton	Australia	Lambeth et al., 2016b
mCherry expressing chickens	Technical University Munich, School of Life Sciences Weihenstephan, Department of Animal Sciences, Reproductive Biotechnology	Germany	Trefil et al., 2017
	BIOPHARM, Research Institute of Biopharmacy and Veterinary Drugs and Institute of Molecular Genetics of the Czech Academy of Sciences	Czech Republic	
*DDX4* KO chickens	The Roslin Institute and Royal Dick School of Veterinary Studies, University of Edinburgh	UK	Taylor et al., 2017
3D8 single chain variable fragment (scFv) expressing chickens	Animal Biotechnology Division, National Institute of Animal Science and Department of Avian Disease Laboratory, College of Veterinary Medicine	South Korea	June Byun et al., 2017
Chickens with humanized immunoglobulin genes	Crystal Bioscience/ Ligand Pharmaceuticals Inc	USA	Ching et al., 2018
Chickens overexpressing human IFN-β	Biomedical Research Institute, National Institute of Advanced Industrial Science and Technology	Japan	Oishi et al., 2018

Specific gene editing in PGCs was improved using TALEN and CRISPR/Cas9 via HDR (Dimitrov et al., 2016; Oishi et al., 2016; Taylor et al., 2017). Using CRISPR/Cas9, the efficiency of gene targeting was increased remarkably in PGCs (Dimitrov et al., 2016). In order to introduce a loxP site into the immunoglobulin heavy chain locus, Dimitrov and colleagues combined a targeting vector having a total of 2 kb homology arms with CRISPR/Cas9 system targeting the upstream region of the single immunoglobulin heavy chain variable region (VH) in PGCs (Dimitrov et al., 2016). Interestingly, all selected drug resistant PGC clones contained the correct targeting event and the germline transmission rate varied between 0 and 100% depending on the used PGC line (Dimitrov et al., 2016).

Targeting the *DDX4* locus, located on the Z chromosome, showed possible role of this gene in the formation of the germ cell lineage (Taylor et al., 2017). Targeted *DDX4* KO was achieved with TALEN in combination with a targeting vector. Authors reported a germline transmission rate of 6% from the founder birds (Taylor et al., 2017). G1 female chicks were hemizygous mutant for *DDX4*, they did not lay eggs and had no yellow or white follicles in the ovaries. Surprisingly this was not the case in *DDX4* knockout female mice (Tanaka et al., 2000).

Overall, a significant progress was made in the last decade in producing and using genetically modified chickens to understand developmental biology, immunology, host-pathogen interaction, reproductive biology and physiology. However, efforts to generate resistant chickens for specific pathogens are still at the beginning, probably due to the lack of specific gene targets responsible for acquiring resistance against specific pathogens. This was not the case in other livestock including pigs which were genetically edited to gain resistance against porcine reproductive and respiratory syndrome virus (PRRSV) (Whitworth et al., 2015; Burkard et al., 2017). Using NHEJ, Whitworth and colleagues generated KO pigs with premature stop codon in exon 3 of the viral receptor CD163 (Whitworth et al., 2015). CD163-KO pigs challenged with PRRSV did not exhibit any clinical symptoms, lung pathology, viremia, or antibody response. In addition, Burckard and colleagues generated an exon 7 deletion in CD163 using two sgRNAs to induce the excision of the exon (Burkard et al., 2017). Pigs carrying the mutation were healthy and kept the main biological functions of the protein while macrophages isolated from the CD163 KO animals indicated an inhibition of the viral infection (Burkard et al., 2017).

So far, only few reports are available about the resistance of gene-edited chickens for specific pathogens. Lyall and colleagues generated transgenic chickens expressing short-hairpin RNA intended to function as a decoy that interacts and blocks influenza A virus polymerase (Lyall et al., 2011). Although birds were not resistant to initial infection, viral transmission was prevented (Lyall et al., 2011). A different study demonstrated the possibility to suppress influenza A virus transmission in transgenic birds expressing the 3D8 single chain variable fragment (scFv), a gene that interacts with viral genome leading to suppression of viral shedding (June Byun et al., 2017).

## Further applications in biomedical research

The chicken became a very interesting model in biomedical research. Different temporal patterns of bright light were used to study the effect on myopia in chickens. Lan and colleagues found that intermittent episodes of light suppress myopia in chickens more than continuous bright light (Lan et al., 2014). Although the obtained results may not be directly translated into humans (Lan et al., 2014), future applications in optical research seem to be promising. In addition, the chicken was used as a model for xenotransplantation by injection of human stem cells into small induced lesions in the chicken embryo neural tube (Boulland et al., 2010). Authors stated that the reduced immune response during early embryonic development helps to study xenotransplantation without the risk of early immune rejection (Boulland et al., 2010). The chicken was also used as a human multiple myeloma xenograft model (Martowicz et al., 2015); it was suggested that this model may offer novel therapeutic compounds targeting survival and proliferation of multiple myeloma cells. Using the chicken as a bioreactor may greatly benefit human health by providing alternative therapeutic approaches (Zhu et al., 2005). A promising approach using chickens for the production of human antibodies is the replacement of the chicken immunoglobulin variable regions by human V regions and synthetic pseudogene arrays in order to produce affinity matured human antibodies in chickens (Ching et al., 2018). The OmniChicken by Ligand Pharmaceuticals Inc. is a worldwide unique platform to produce human monoclonal antibodies from chickens making use of the phylogenetic difference between mammals and birds. The purification of overexpressed human antibodies from the chicken egg seems also to be a valid application which was reviewed elsewhere (Flemming, 2005). A very recent study conducted by Oishi and colleagues demonstrated the ability of integrating human interferon beta (hIFN-β) into the chicken ovalbumin locus in order to produce hIFN-β in egg white (Oishi et al., 2018). Authors demonstrated the ability of producing foreign proteins in eggs which would have industrial and therapeutic applications.

## Future perspectives

The role of host genes in the susceptibility of chickens to different pathogens was mostly investigated *in vitro*. Preliminary *in vitro* investigations provide solid information about the role of these genes prior to the generation of fully gene edited chickens. New technologies including CRISPR/Cas9 make the process of gene editing easy and highly efficient in contrast to the well-established process of homologs recombination. Although gene editing in mammals, particularly mice and pigs, is vastly advanced, gene editing in chickens is entering the golden age. For instance the generation of Cas9-expressing pigs will provide a powerful tool for the study of biological processes (Wang et al., 2017); while this was not done yet in chickens, it seems to be beneficial and may be used in the future to dissect unknown gene functions faster and more easily.

Therapeutic applications using human monoclonal antibodies produced from humanized chickens may be beneficial over *in vitro* approaches lacking affinity maturation (Ching et al., 2018). In addition, production of antibodies in chicken eggs represents an economic and stress-free method for the production of specific antibodies (Amro et al., 2018). Using chicken eggs to manufacture specific proteins in eggs seems interesting (Lillico et al., 2005; Petitte and Mozdziak, 2007) especially since it may allow improvement of digestibility of sugar complexes in feedstuffs; however, this application may be thwarted by critics that claim the inedibility of the product.

Several advantages are provided by newly invented gene editing technologies including the simplicity of design and application combined with high efficiency (Chira et al., 2017). Understanding the host cell behavior during host-pathogen interactions may help targeting pathogen specific receptors and viral cellular transport (Heaton et al., 2016). The determination of new target genes associated with disease susceptibility should fill the research gap and open the door for new therapeutical approaches. Although the debate about using genetically modified animals in food production will continue to be stimulated, we may obtain new breeds of chickens in the future that are resistant for specific pathogens. We speculate that spending more efforts connecting gene editing technologies with the prevention of infectious diseases will change the way we use to fight pathogens and will probably improve the animal welfare.

## Author contributions

All authors listed have made a substantial, direct and intellectual contribution to the work, and approved it for publication.

### Conflict of interest statement

The authors declare that the research was conducted in the absence of any commercial or financial relationships that could be construed as a potential conflict of interest.
